# Schistosoma Cattoi : A New Blood Fluke of Man

**Published:** 1905-01-14

**Authors:** 


### SCHISTOSOMA CATTOI: A NEW BLOOD FLUKE OF MAN.

ANOTHER discovery in tropical medicine formed the subject of the Cragg's Prize Essay for 1904, which was awarded to Mr. John Catto. Mr. Catto found, while examining the viscera of a Chinese coolie who had died of cholera, certain conditions which led him to make a minute examination of the tissues, with the result that he discovered trematode worms in the vessels of the mesocolon, and their ova in the mucosa and villi of the large intestine. The parent worms, which do not appear to be identical with any known species, were present in small groups at the bifurcations of the smaller mesenteric vessels, while the ova for the most part occupied two concentric layers in the coats of the large intestine—namely, the sub-peritoneal layer, where they were relatively scarce, and the submucous layer, where they were innumerable. Where the ova were accumulated they had caused chronic inflammation, so that there were signs of chronic peritonitis and ulceration of the intestinal mucosa. Ova were also present in the mesenteric glands and liver which were enlarged. Mr. Catto suggests that the ova of this parasite may have been previously seen many times in the course of microscopical examinations of the fæces and have been mistaken for the ova of ankylostoma duodenale, which they closely resemble. The symptoms of this infection are probably colic and dysentery with enlargement of the liver and spleen.

